# Mechanical and tribological performance of a hybrid MMC coating deposited on Al–17Si piston alloy by laser composite surfacing technique

**DOI:** 10.1039/c7ra08191j

**Published:** 2018-02-13

**Authors:** M. M. Quazi, M. Ishak, A. Arslan, M. A. Fazal, Farazila Yusof, B. S. Sazzad, M. Nasir Bashir, M. Jamshaid

**Affiliations:** Department of Mechanical Engineering, Faculty of Engineering, Universiti Malaysia Pahang Pekan 26600 Malaysia engrmoinquazi@gmail.com +60-192048281; Department of Mechanical Engineering, Faculty of Engineering, University of Malaya 50603 Kuala Lumpur Malaysia; Department of Mechanical Engineering, University of Jeddah Saudi Arabia; Department of Mechanical Engineering, International University of Business Agriculture and Technology Dhaka Bangladesh

## Abstract

Laser composite surfacing (LCS) is a photon driven manufacturing technology that can be utilized for depositing hybrid metal matrix composite coatings (HMMC) on softer Ti/Al/Mg alloys to enhance their tribo-mechanical properties. LCS offers the advantages of higher directionality, localized microstructural refinement and higher metallurgical bonding between coating and substrate. The current research presents the tribo-mechanical evaluation and characterization of solid lubricant based Ni–WC coatings deposited by LCS on Al–Si piston alloy by varying the concentration of graphite between 5-to-15-weight percentage. The tribological behavior of LCS samples was investigated using a ball-on-plate tribometer. Results indicate that the surface hardness, wear rate and friction coefficient of the Al–Si hypereutectic piston alloy were improved after LCS of graphite based HMMC coatings. The maximum surface hardness of 781*H*_v_ was acquired for the Ni–WC coating containing 5 wt% graphite. The friction coefficient of Al–Si under dry sliding conditions was reduced from 0.47 to 0.21. The reduction in the friction coefficient was attributed to the formation of a shearable transfer layer, which prevented delamination and reduced adhesion, abrasion and fatigue cracking.

## Abstract 2

The addition of graphite as solid lubricant has significantly reduced the friction coefficient and wear of a Ni–WC composite coating.

## Graphical abstract

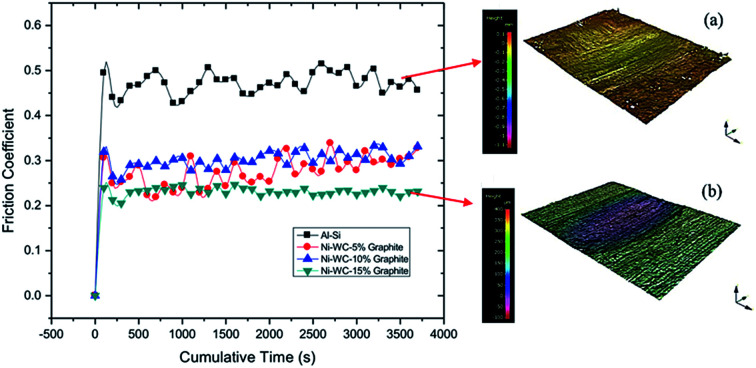

## 1. Introduction

Rapid progress in automotive and aerospace industries is now driven by incorporation of novel advanced technologies, development of new material systems and implementation of the latest manufacturing technologies which can help reduce vehicle energy consumption, and component service lifetimes at a competitive cost. One way of achieving this goal is by replacing high-density ferrous bulk materials with high strength aluminium (Al) alloys. However, the challenge lies in employing Al alloys in a harsh tribological system that is a part of an aggressive system in I.C. engines wherein synergistic properties such as high-temperature wear and self-lubricating properties are required. Replacing cast iron engine components with lightweight Al alloys requires overcoming the poor adhesion and seizure resistance of Al under a hostile environment. Employing Al alloys in engine components, such as in pistons sliding against cast iron liners, produces higher wear and friction.^1^

There are various surface modification techniques available including electrodeposition,^2^ physical vapor deposition,^3^ surface texturing^4^*etc.* Amongst available techniques, one technique to improve tribological properties of Al surfaces is to introduce laser controlled melting at the surface in the presence of a composition modifying and particulate embedding materials.^5^ Laser surface modification offers the added advantage localized processing, and only regions on the surface that require protection against wear are needed to be processed thereby, creating improved surfaces having the high strength metallurgical bond to the substrate. This high strength bond is an asset when considering the tribological properties of the modified surfaces since defects at the interface between a wear-resistant coating and its substrate is known to lead to delamination failure.^6^ With the assistance of lasers technology, composite layers can be fabricated on Al alloys to enhance their tribological performance.^7^

The addition of ceramic particles such as silicon carbide (SiC) often leads to the dissolution of the carbides resulting in Al_4_C_3_ that are unwanted due to their negative effect on mechanical properties. Tungsten carbide (WC) has higher hardness and toughness, good radiation absorption and is stable at high temperature.^8^ It does not dissociate significantly during the laser melting process, as in the case of SiC. However, reinforcing hard ceramic particulates like borides, carbides and nitrides may improve the tribological properties of substrates by increasing surface hardness but subsequently produce high friction coefficient.^9^ To overcome this difficulty, hybrid metal matrix composites (HMMCs) can be fabricated with the addition of self-lubricating particulate phase.^10^ When solid lubricants enter the gap between interacting surfaces in relative motion, they achieve lubrication by accommodating the relative surface velocities by easily shearing; coalesce in the surface asperities; carry a portion of the asperity contact load.

In our review of laser surface modification of Al alloys,^11^ it has been noted that high strength wear resistant metal matrix composite coatings are obtained when nickel in combination with carbide phases are added in Al alloys resulting in the formation of Al–Ni intermetallic phase that possesses greater wear resistance. But, the literature points out that researchers have been unsuccessful in studying the frictional characteristics of the laser claddings deposited on Al substrates and some of the research carried out have indicated high friction coefficient owing to the nature of the composite coating. In another comprehensive review on laser cladding of self-lubricating wear resistance composite coatings,^12^ we have further emphasized the beneficial aspects of solid-lubricants in reducing the friction and wear of laser claddings deposited on steel and titanium substrates. There exists a significant gap in realizing the potential of solid lubricants in enhancing the friction and wear of Al alloys with the fabrication of self-lubricating wear resistant composite coatings. The present study is aimed to prepare a hybrid composite Ni–WC coating with graphite serving as self-lubricant in the coating deposited on Al–Si piston alloy by laser composite surfacing so as to investigate hardness and tribological behaviours under dry wear regime.

## 2. Experimental procedures

### 2.1. Material preparation

In this research work, hypereutectic Al–Si piston alloy was investigated comprising of a chemical composition consisting of 0.11% Zn, 0.07% Mn, 1.64% Cu, 0.06, 18% Si, 0.09% Cr, and Al balance (in wt%). Samples were cut into square samples of 6 mm thickness and 15 mm width with electric discharge wire cutting machine. Thereafter, prior to laser processing, the controllable parameter, which is surface roughness of Al–Si alloy, was increased to 2 μm by sand blasting. Not only does sand blasting increases the incidence beam absorption, but also it removes the oxide scales and enhances pre-placed powder adhesion. Without dispersing and removing the oxide film, may result in the formation of a molten pool of Al enclosed in a skin of oxide and may cause additional porosity.

In order to avoid excessive melting of the substrate, alloying powder with higher melting points were pre-placed onto the substrate for composite surfacing as the pre-placed powder layer exhibit far better absorption of laser energy than solid Al because of the pores present in the layer. Pre-placed powder layer due to its porous structure exhibit low thermal conductivity and major portion of incident beam energy is absorbed in the layer guaranteeing unnecessary melting of the substrate. The powder mixtures for laser composite surfacing, composed of 56 wt% of Ni and 44 wt% of WC. Raw powder size used were 1–10 μm for tungsten carbide (WC) from Sigma-Aldrich (assay ≥ 99%) and 75–150 μm for nickel (Ni) powders from Wako (assay ≥ 99%). Raw powder sizes used for graphite (Gr) were in the range of 100–150 μm from Sigma-Aldrich (assay ≥ 99%). The powder to be pre-placed in proportion was mixed with 5 wt% organic binder poly vinyl alcohol (PVA, P1763, Sigma Aldrich). The binder slurry was made by mixing 5 wt% PVA with 95 wt de-ionized water and magnetically stirred for 4 hours on a hot plate maintained at a temperature of 100 °C. Once, the slurry like consistency was obtained, the coating powders were mixed and was applied onto the sample with a pre-deposition thickness of around 100 to 200 μm. The binder was thereafter dried in the furnace (80 °C for 2 hours).

### 2.2. Laser surface processing

A 300 W, ytterbium (Yb) doped fiber-optic laser (Rofin Starfiber 300) is operated at a wavelength of 1070 nm in continuous wave mode. By employing ZnSe lens with focal length of 350 mm and the smallest beam spot size of 79 μm, the laser beam was defocused onto the samples. The beam parameters were set in defocused condition as larger melt pools develop at negative defocusing at a given spot size and heat input than for positive defocusing. Transverse electromagnetic mode (TEM_00_) beam with an intensity profile of a Gaussian distribution was utilized to propagate the beam with an overlapping of 20%. Fiber lasers have typical beam quality *M*^2^ value of less than 1.05 mm rad. This excellent beam characteristic sets apart Yb doped fiber lasers from CO_2_ and Nd: YAG lasers for achieving higher power densities with beam focusing on much smaller irradiation areas. For an applied power of 300 W, a max power density of about 6.12 MW cm^−2^ can be obtained. After a series of experiments to obtain defect free coating, the laser power of 150 W, defocus distance of 4 mm and scanning speed of 12 mm s^−1^ were selected for all the experiments performed.

### 2.3. Tribological testing

The wear response of laser composite surfaced samples was compared to that of the as-received Al–Si by a high-frequency linear-oscillation “DUCOM ball-on-plate reciprocating friction monitor TR-282” under the dry sliding condition. The experiments were carried out under normal loads of, 20 N with a reciprocating frequency of 10 Hz and amplitude stroke of 2 mm for 30 minutes. The total sliding distance measured by machine software was 144 meters. Besides, for weight loss measurement, a high precision weight balance “Denver Instrument” with an accuracy of 0.1 mg was utilized in order to calculate the wear. Steel counter-body was utilized in this study.^13^ The counter-body balls of 6 mm diameter were obtained from AISI 440C quenched and tempered tool steel (800*H*_v_) containing C 1.2 wt%, Cr 16 wt%, Mn 1 wt%, Si 1 wt%, Mo 0.75 wt%, S 0.030 wt%, P 0.040 wt% and Fe balance. The laser treated samples were grounded after surface treatment to produce an acceptable smooth surface. Before commencement of wear testing, samples and balls were washed ultrasonically in acetone for 10 min.

### 2.4. Metallography and characterization

For optical and scanning electron microscopy, LCS samples were sectioned, polished, ultrasonically cleaned in an acetone bath. Olympus BX 61 was used for light optical microscopy (OM). The surface morphology, cross-sections and elemental characterizations were investigated by scanning electron microscope (SEM) using “Hitachi 3400N” equipment integrated with energy dispersive X-ray spectroscopy (EDX). EDX quantitative analysis was performed on at least two different locations on each sample so as to ensure that the measurements were indicative of the entire microstructure as well as to assess the degree of sample variability. The phase formation of the coating was investigated by X-Ray Diffraction (XRD) analysis using an “Empyrean” X-ray diffractometer with a Cu-source (*λ* = 0.15406 nm) using a Bragg–Brentano configuration, step-scanned in the 2*θ* range of 20–80° with a scan rate of 0.1° s^−1^ and step size of 0.026°. The micro hardness of the composite layer was measured on the top surface as well as along the cross-sectional plane by using Vickers micro-hardness tester “HMV Micro Hardness Tester Shimadzu”. Minimum 3 tests were carried out across each sample and the average values were collected. The duration of the hardness tests was set to 20 seconds and the applied load was 980.7 mN (*H*_v0.5_). All tests were performed in laboratory ambient temperature of 28 °C. “Alicona 3D optical” microscope was used for 3D scanning of the worn surface.

## 3. Results, analysis and discussion

### 3.1. Microstructure & phase identification

**Fig. 1:**
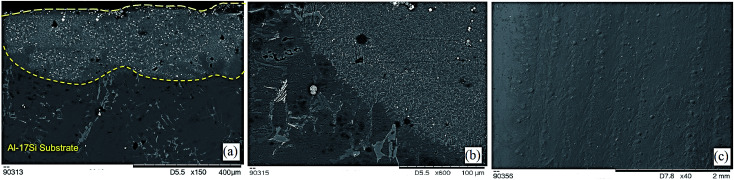
SEM cross-sectional view of laser composite surfacing of (a) Ni–WC–graphite coating, (b) substrate/coating interface, (c) overlay of Ni–WC–graphite coating.

The cross-sectional SEM micrograph of Ni–WC-5 wt%–Gr coating deposited on Al–17Si substrate is showed in Fig. 1(a). The interface between coating and substrate is clearly visible with no signs of cracks. Simultaneous tracks with an overlapping percentage of 20% were scanned to obtain a coating thickness greater than 200 μm with each overlay being 400 μm wide. Besides, Fig. 1(b) shows the cross-sectional substrate to coating interfaces. Albeit, some forms of porosities were inevitable. SEM overview of the Ni–WC-5 wt%–Gr coating laid on Al–17Si alloy is also given in Fig. 1(c).

**Fig. 2:**
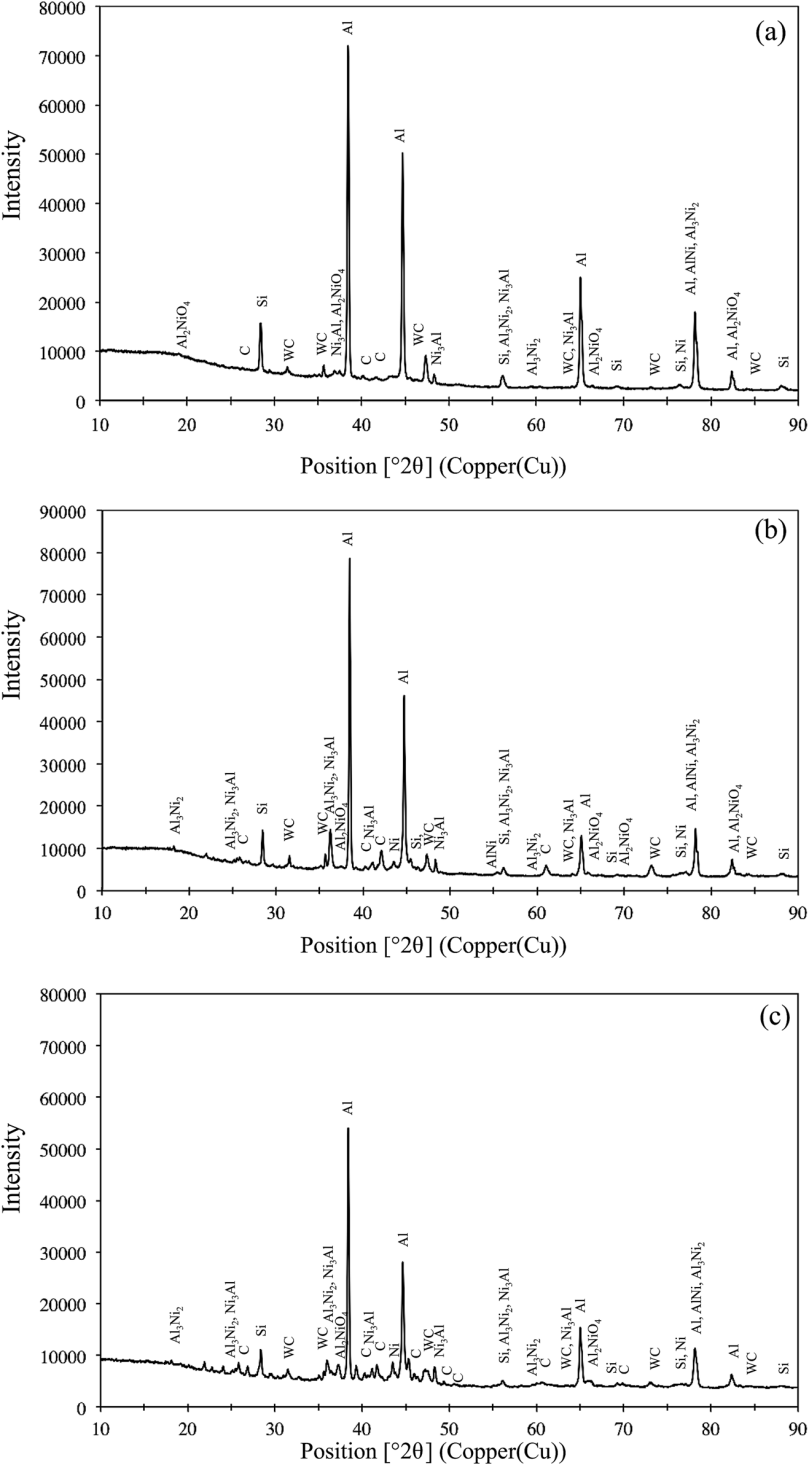
The X-ray diffraction spectrum for Ni–WC coating with graphite content (a) 5 wt% (b) 10 wt% and (c) 15 wt%.

The X-ray diffraction pattern of Ni–WC–Gr composite coating with varying concentration of Gr content is presented in Fig. 2. Since the 2*θ* scan was carried out on the metallographic sample cross-section, hence visible higher intensities peaks of Al and Si are present. The coating is composed of WC phase embedded in the Al–Ni intermetallic phase. The intermetallic phase was formed based on the concentration of the nickel in the coating comprising of AlNIi, Al_3_Ni, Al_3_Ni_2_, Ni_3_Al and an oxide form of nickel aluminide Al_2_NiO_4_ was found to be present. It is observed that characteristics reflection of the graphite phase in the form of carbon (C) is also present which increases in intensities as graphite content is raised as depicted in Fig. 2(a)–(c). Some of the reflections comprise of double peaks or peaks with shoulders wherein multiple compounds are formed are also present. The addition of untransformed graphite has been also reported in the open literature.^14^

### 3.2. Surface hardness characterization

**Fig. 3:**
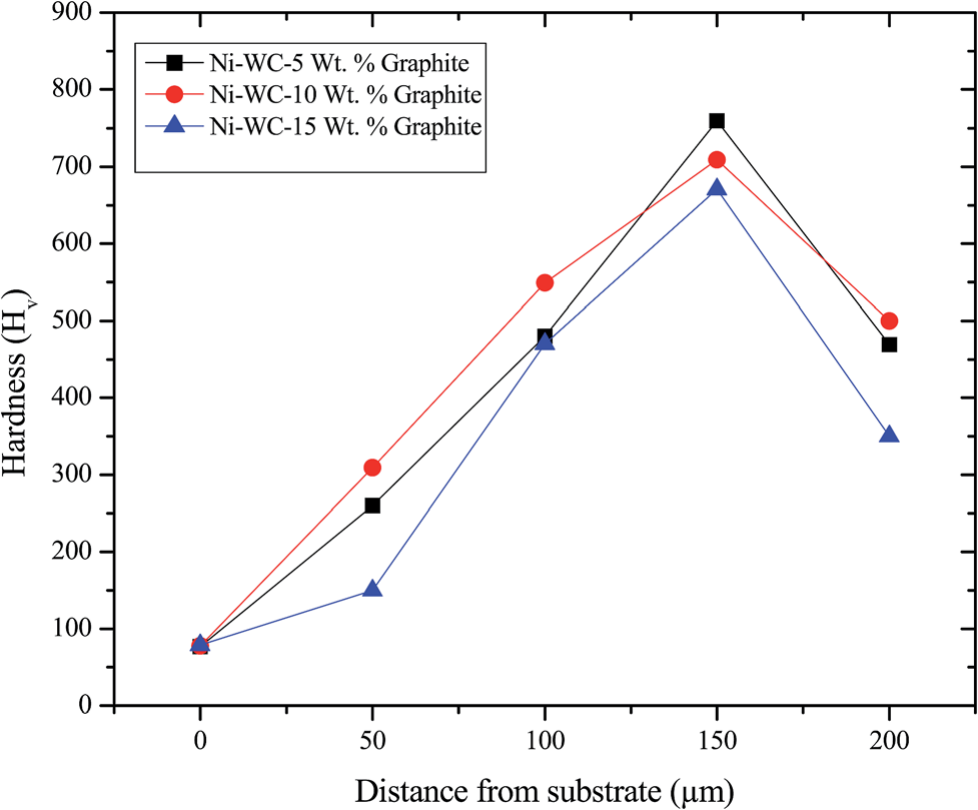
The surface hardness profiles for Ni–WC coating with graphite.

The hardness as measured on the cross-sections of the 200 μm thick MMC and HMMC coatings are provided in Fig. 3. The profile starts from substrate hardness and then as the concentration of nickel increases, it enters in the eutectic zones. The hardness of graphite based coatings decreases as the concentration increases with a maximum hardness value for Ni–WC-5 wt%–Gr being 781*H*_v_. As the percentage of Ni and WC reduces towards the end of coating, the hardness of substrate is eventually reached. Graphite being softer and less hard reduces the overall hardness of the composite whose properties depend on the volume fraction of each constituent added. Further to this, the hardness of the respective phases of Al–Ni, Ni, WC and graphite itself contribute to the overall strengthening mechanism. Choudhury *et al.*^14^ fabricated nano based MMC coating containing TiB_2_, TiB and graphite phase on steel specimens and reported surface hardness ranging from 800 to 2000*H*_v_.

### 3.3. Wear and friction coefficient of substrate and coatings

**Fig. 4:**
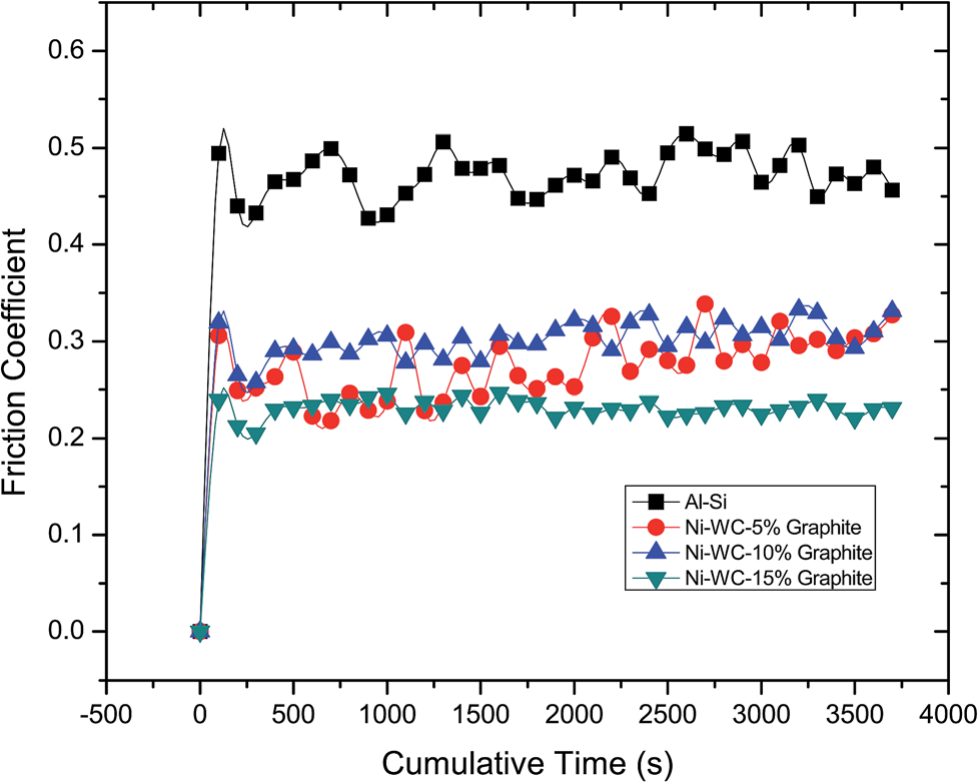
The friction coefficient of Al–17% Si and coatings with graphite.

The friction coefficient *versus* sliding distance for Al–17% Si and Ni–WC coating with additives containing various concentrations is displayed in Fig. 4. Friction coefficient of Al–17% Si did not attain a steady state condition, which continued to rise towards the end of the experiment with the greater number of fluctuations. A notable feature observed in the initial period of all tests that were conducted was higher friction coefficient values. Higher friction was attributed to running in wear, characterized by the conformity of worn surfaces and initial successive wear of surfaces' micro asperities. Therefore, in the early stages of wear, the damage is caused by micro-fractured brittle fragments within surface grains along with the removal of the oxide layer and changes occurring in chemical composition. Thereafter, higher and stable friction in the later stages is conceded by tribochemical reactions that drastically contributes to the total amount of wear ensued. The running in wear process is acceptable wear regime experienced by components in the earlier operating stages of their lifetimes. Higher friction coefficients values at lower solid lubricant concentrations for Ni–WC coating is indicative that the amount of additives added was not enough for the transfer layer to form. Albeit, by increasing the concentration of additives, a significant reduction in friction coefficient, is observed.

**Fig. 5:**
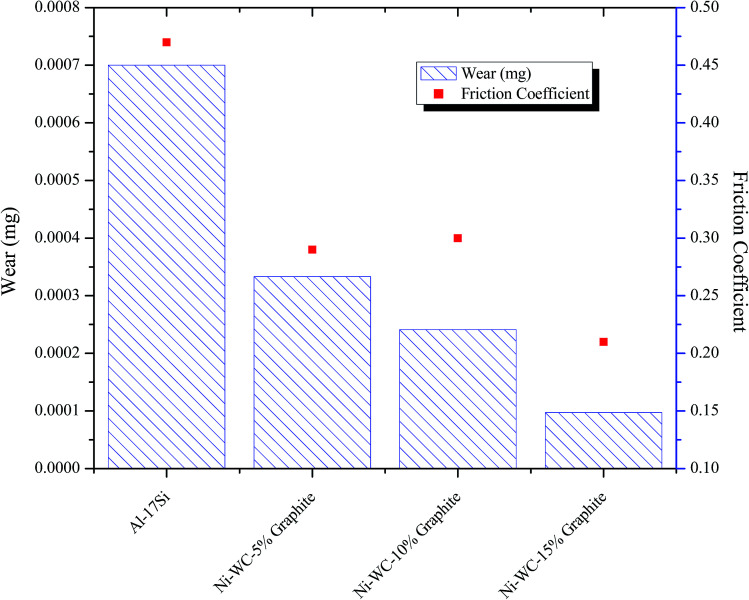
Wear response and steady state friction coefficient of Al–17Si and laser treated Ni–WC coating with additives.

The variation of the steady-state friction coefficient as a function of graphite concentration is depicted in Fig. 5. In general, a decrease in friction coefficient with respect to the normal load is detected. The mean value of friction coefficient was found to be 0.47 for Al–17Si, 0.29 for Ni–WC-5 wt%–Gr, 0.30 for Ni–WC 10 wt%–Gr, 0.21 for Ni–WC 15 wt%–Gr. The lower average friction coefficient of the coatings can also be due to the microstructural and grain refinement as smaller grain exhibits lower friction coefficient values in contrast to the base alloy, which contains different volume fraction and composition of the alloying elements. Al–17Si alloy exhibited a friction coefficient value of 0.47, which has been observed for dry sliding friction of Al–Si alloys. This is typical of the values of coefficient of friction in the dry sliding wear of Al–Si sliding against the harder steel counterpart.^15^ This higher value associated with Al–Si alloys has been attributed to the impact of the specific sliding condition leading to higher friction being generated. At lower sliding speeds, the more extent of the time period is available for the growth and evolution of asperity contact regions. This increases the amount of frictional force required to shear away these asperity contacts so as to maintain the contact relative motion.^15^

For graphite based coatings Wang *et al.*^16^ inspected the impact of graphite composition (0 to 10%) on the wear and friction characteristics of Zr–Al–Ni–Cu cladding deposited on titanium substrate. They found that the addition of 5% graphite was beneficial for producing higher hardness and minimum friction coefficient in the range of 0.20–0.27. Similarly, Ye^17^ reported that with the addition of 15 wt% graphite, friction coefficient in the range between 0.2 to 0.25 is achievable. Scharf & Prasad^18^ also observed the friction coefficient values in the range of 0.21–0.22 for graphite based hybrid metal matrix coatings. Choudhury *et al.*^14^ showed that for nano based MMC coating containing TiB_2_, TiB and graphite phase deposited on steel specimens a reduction in friction coefficient from 0.6 to 0.37 is observed. In one study it was found that the wear rate was correlated with the concentration of graphite in the plasma sprayed metal matrix composite coating at a maximum of 8 wt% graphite.^19^ The beneficial effects of graphite were also explored by Yang *et al.*^20^ where graphite was added in the Ni-60 super hard alloy to realize a significant improvement in friction coefficient from 0.55 to 0.20. Ying *et al.*^21^ fabricated Ni60/TiC/graphite coating for stainless steel specimen to witness a drop of friction coefficient from a range of 0.7–0.8 to that of 0.4–0.5.

The relation of wear and friction is further corroborated in Fig. 5 showing a variation of wear with concentration. For nearly all surfaces tested, the wear resistance of the treated samples was considerably better than that of the untreated. The wear of coating, containing 15 wt% graphite as an additive was considered to be almost 7.2 times lower than the bulk material samples. In the open literature, Tian *et al.*^22^ when silicon and graphite were added as additives in laser cladding produced on Ti–6Al–4V, an enhancement in wear resistance to about 5 times and reduction in friction coefficient 0.5 to 0.37 is realized. Zhang *et al.*^23^ prepared *in situ* laser cladding comprising of TiC, TiN, TiB and TiB_2_ phases by the using powder composition of B_4_C and 40–50% graphite. With graphite in excess, a decrease in wear to about 3 to 5 times was seen. Other manufacturing processes are already reaping the benefits of entraining the solid lubricants in their respective material system. For instance, laser engineered net shaping (LENS) has been employed to fabricate self-lubricating wear resistant metal matrix coatings and owing to their industrial worth is now patented.^24^ In addition, the material systems that comprises of the solid lubricants have considerable value and are being patented.^25^

### 3.4. Wear characteristics of substrate Al–17Si alloy

#### 3.4.1. Characterization of worn surfaces

**Fig. 6:**
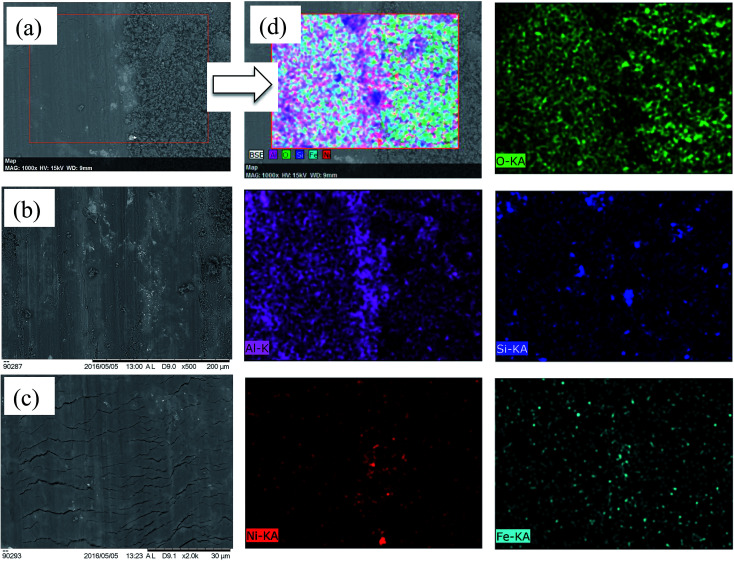
Scanning electron micrograph of worn surfaces for Al–Si alloy (a) debris–scar interface, (b) and (c) at 0.5k and 1.0k magnification along with corresponding (d) EDX elemental mapping of O, Al, Si, Ni and Fe elements.

The wear of the Al–17% Si hypereutectic piston alloy at 20 N applied normal load is showed in Fig. 6. Fig. 6(a) shows that the wear of Al–Si alloy was characterized by the considerable generation of wear debris in the form worn out particles. These particles that were formed on the wear scar were found to be higher in the content of oxygen as presented in Fig. 6(d). They contained certain amounts of iron, silicon, nickel and aluminium. As illustrated in Fig. 6(b) abrasion in the form of grooves and scratches are also visible which are translated into transverse cracks as wear progress. The crack propagation progressed until rapid material removal in the form of plate-like debris. The harder asperities formed on stainless steel counter-body shifted the material to the side of the grooves on wear scar. These grooves (30–40 μm) were formed due to smearing and ploughing action, which are characteristics of severe plastic deformation. Due to the applied reciprocating cyclic load, fatigue cracks were generated during the wear process, and delamination started which indicated the initiation of debris formation in the transfer layer. The cracks that are propagated connect with each other and the fragmented metallic particles are further fragmented to form mechanically mixed oxide particles in the contact zone. During wear when tribo film is generated, it is further weakened when the stresses derived on the sliding surface leads to the fracture of oxide film to produce oxidized debris.

#### 3.4.2. Counter-body wear and debris analysis

**Fig. 7:**
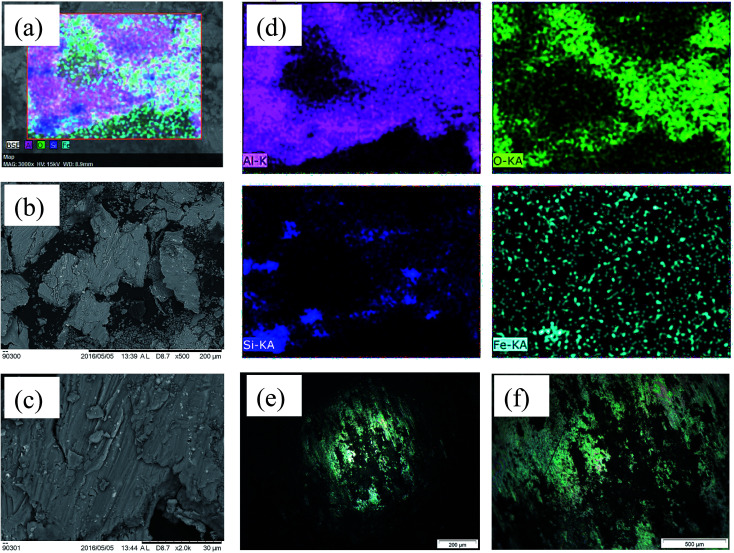
(a) EDX mapping of images for debris of Al–17% Si debris elemental mapping, (b) and (c) SEM image of debris at 0.5k and 2.0k magnification, (d) EDX elemental mapping of Al, O, Si and Fe and Fe element debris, (e) and (f) optical micrograph of counter body scar at distinctive magnifications.

Fig. 7(a)–(d) shows the SEM images of debris and their EDX area mapping along with OM of counter-face. Fig. 7(e) and (f) shows the presence of oxidized material transferred from counter-body further deteriorating the wear of Al–Si alloy. At the beginning of wear tests, small pieces of material were pulled off that adhered to the counter-body. Thereafter, subsequently rubbing action oxidizes the adhered material that correlated with the EDX analysis. These oxidized elements might be trapped between the sliding surfaces and get crushed or compacted due to the repetitive sliding. It can be clearly illustrated that severe abrasive wear with fatigue cracks characterized the wear mechanism for Al–Si alloy. A similar explanation was given for severe wear of Al–Si alloys^26^ wherein it was pointed out that wear mechanism of Al–Si alloy against stainless steel, revealed an intense delamination, abrasion and adhesive wear.

The plate-like delaminated Al debris and the iron-transferred debris further assisted in the greater material removal as presented Fig. 7(b) and (c). The oxide particles were generated as presented in the EDX of the wear debris (Fig. 7(a) and (d)). The microstructure at higher magnification shows severe plastic deformation in terms of deeper and wider grooves that are also present on the worn-out debris. Under severe wear regime in the case of Al–S alloy, debris generated was in the form of larger plate-like fragments (100–200 μm) in addition to smaller equiaxed particles (0.5–20 μm). Sheets like debris are a sign of delamination wear mechanism.^27^ This is also confirmed in scar morphology of counter-body where the sheared and oxidized material is transferred and adhered onto the scar surface (Fig. 7(e) and (f)).

### 3.5. Wear characteristics of Ni–WC–graphite coating

#### 3.5.1. Characterization of worn surface

**Fig. 8:**
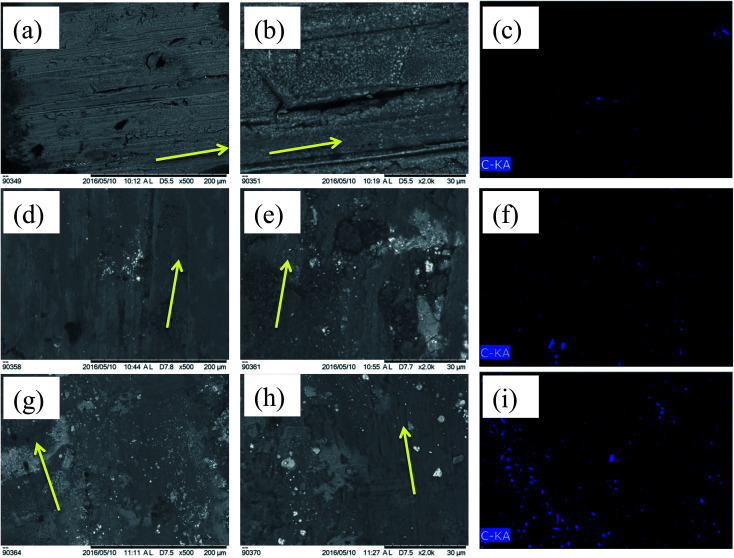
SEM image of coating wear with their magnified views for (a), (b) 5 wt% graphite (d), (e) 10 wt% graphite, (g), (h) 15 wt% graphite, along with their corresponding EDX carbon element mapping in (c), (f) and (i).

The worn surface morphology and EDX area mapping (element-C) of Ni–WC–Gr coating deposited on hypereutectic piston alloy under an applied load of 20 N are depicted in Fig. 8. Fig. 8(a) and (b) shows that the wear of Ni–WC-5 wt%–Gr was generalized by the formation worn groove and light scratches. As illustrated in Fig. 8(b), abrasion in the form of grooves and scratches are visible which were translated into parallel cracks to the sliding direction. These fatigue cracks started material removal, in the form of adhered or compacted debris removal. These grooves (30–40 μm) were formed due to slight smearing action. As a result of reciprocating cyclic load, fatigue cracks may have been generated. At higher frequencies and temperature, micros cracks are said to initiate which propagated due to the involvement of fatigue induced wear. The EDX area mapping in Fig. 8(a)–(c) depict that as the amount of graphite concentration raises, the severity of the corresponding wear scars on the coating reduces.

EDX area scan on worn scar morphology for Ni–WC–graphite coatings was carried out with a change in graphite content. It was observed that the amount of carbon detected grows strongly as with concentration and so does the oxidation in the form of oxide formation. The intensity and formation are abrasives are reduced as the addition of graphite assists in the formation of an effective transfer layer that may shear itself away inform of oxidized mixtures of various coatings and counter-face materials that are ejected during wear inform of wear debris. These softer phases negate the cutting effect of carbide phase and hence assist in the reduction of material loss. The wear mechanism for higher concentrations of graphite seems to be more tending towards slight abrasive and more of an oxidative. It was found in one study^19^ that the addition of graphite improves abrasion resistance and was indicated that the right amount of graphite can impart a sufficient amount of lubricating effect during service lifetimes of the components.

#### 3.5.2. Counter-body wear and debris analysis

**Fig. 9:**
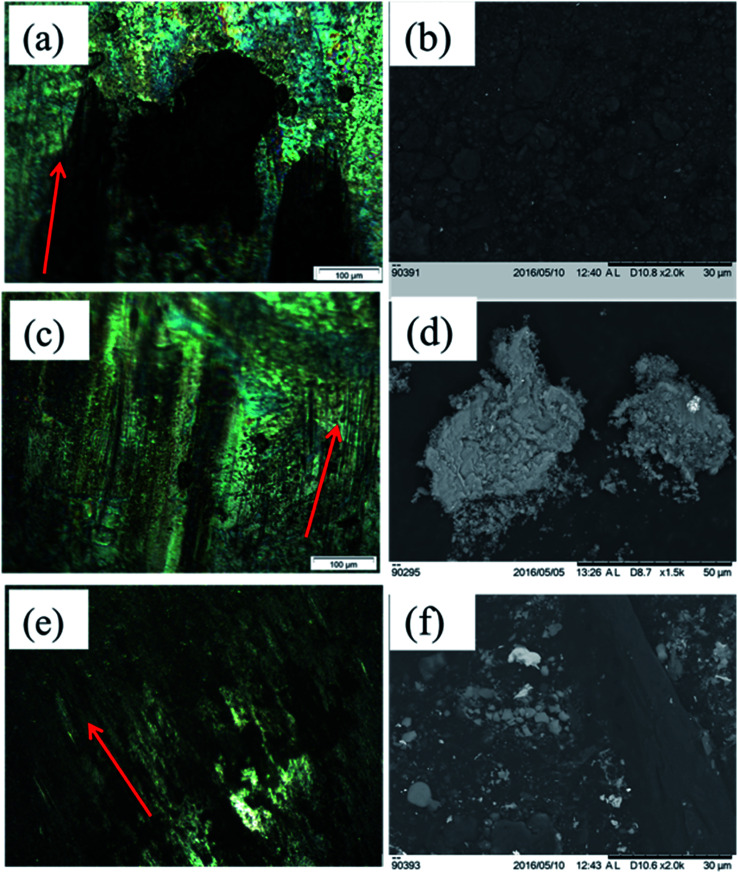
Optical micrographic images of counter-body worn tracks and SEM images of worn debris for (a), (b) 5 wt% graphite (c), (d) 10 wt% graphite, (e), (f) 15 wt% graphite respectively.

The wear mechanism of the worn surfaces is highly correlated with the wear mechanism identified on the surface of the counter-body. Hence, it is quite important to look into the details of the worn surface morphology, material deposits, shearing, tribo film formation, abrasion, or the structure of the scar. From the Fig. 9(a), (c) and (e), it is quite evident that with respect to the change in concentration of the graphite additive, significant changes in the wear mechanism of the counter-bodies are observed. This is an evidence of how closely the concentration of graphite can impact and alter the wearing phenomenon that took place during dry sliding.

These are the characteristics of adhesion in addition to galling wear for 5 wt% graphite coating, wherein the materials adhere from the contacting surface at higher hertizian contact pressure. The ball on plate induces extreme amounts of hertizian contact pressures, thus, the material galls and the galled material thereafter is grooved with the asperities that are formed at a micro level. After adhesion due to higher friction, slipping and tearing of the crystal structure follow the galling. From Fig. 9(a), the galling phenomena were observed for the counter-body of Ni–WC-5 wt% graphite coating. There are highly adhered blackish deposits, which are somewhat shiny and seems melted or welded onto the hardened bearing steel. Even though the balls were cleaned and degreased with acetone in the ultrasonic cleaner for 30 minutes, these deposits remain attached and hence were neither in any form of debris attached nor loosely adhered coating or Al metal. No significant damage by abrasion is observed on this counter-body. Due to adhesion of coating onto the counter-body, the material was stuck on the counter-body as shown in the optical micrograph of the Fig. 9(a). It has been referred in the literature that the material may itself tear off at greater loads friction weld to the counter-surface. The galled material appears as gouged with the torn lump of the material stuck to the surface of the counter-body. This galling is further reduced when the concentration of graphite was increased to 10 wt% and signs of abrasive damage to the counter-body indicating the resistance of the 10 wt% coating to abrasion itself. These black marks can be carbon deposits that are the part of the partial tribo-film formed on the surface. The counter-body of 10 wt% graphite Ni–WC coating presents slight abrasion inform of wider grooves and deep, signifying the significant damage that the coating has caused to the counter-body. There are black patches of oxide or tribo film that may have formed, but mainly the shiny steel and the grooves appearance indicate that the wear was mainly composed of abrasive based phenomenon.

Although galled material is not present, sheared tribo film chunks are also evident. The surface coverage of adhesion and abrasion is both ample enough to allow the greater portion of counter-body being involved in the sliding wear. The blackish areas and un-shiny non-conducive surface the counter-body are depicting carbon deposits or the possibility of the formation or partial formation of a tribo film easily shearable. This depiction may correlate with the least friction coefficient that has been seen previously. There are no signs of wider grooves present in Fig. 9(e) showing material removal rate was comparatively quite lesser and their depth is far lesser than the 10 wt% graphite coating (Fig. 9(c)).

The amount of wear debris generated during dry sliding of Ni–WC coating with a variation of the percentage of graphite concentration is presented in Fig. 9(b), (d) and (f). The morphology, size, color and type of debris are also indicative of the wear mechanism that ensued the sliding of two hard bodies relative to each other. During wear, the material is transferred again and again between the sliding surfaces and is ejected eventually.^28^ Upon closer examination of the quantity of debris generated, it is found that the amount of debris generated is greater for 5 wt% graphite based coating that subsequently reduces as this concentration increases to 10 wt%. This is also indicative of the severity of wear decreasing as the particle size of debris also decreases when the concentration increases. Ferrous whitish particles in higher concentration were found in 5 wt% coating with debris size in the range of 100 μm to few micrometers are present showing that these hard abrasives further assisted in the cutting of the coating to produce abrasive marks as the sliding progresses. Further to this blackish oxidized aluminium debris with a size range of 400 μm indicating greater material removal was observed. The majority of the wear debris as oxidized was equiaxed. The signs of delamination wear or plate-like debris was absent in all coatings when compared with the substrate wear showing that the material removed was progressing through abrasive means. As the concentration of whitish particles decreases, the size of debris removed from the coating also reduces and higher number of equiaxed particles are generated which is more visible in SEM micrographs as presented in Fig. 9(f). It is believed that the iron material detached from the counter-face due to abrasive nature of the carbide reinforcement phase and is oxidized upon transfer film formation. Deuis *et al.*^28^ in their research concluded that the increase in solid lubricant phase in the matrix decreases the micro cutting effect of carbide phase that may lead to fewer generation of third body abrasives. In another study undertaken by Hirschhron & Daver^29^ it was mentioned that as the amount of graphite increases, the size of the debris particles reduces which correlates to a reduction in surface roughness and width of wear grooves.

### 3.6. 3D surface scans of worn scars for Al–17Si, Ni–WC 15 wt% graphite

**Fig. 10:**
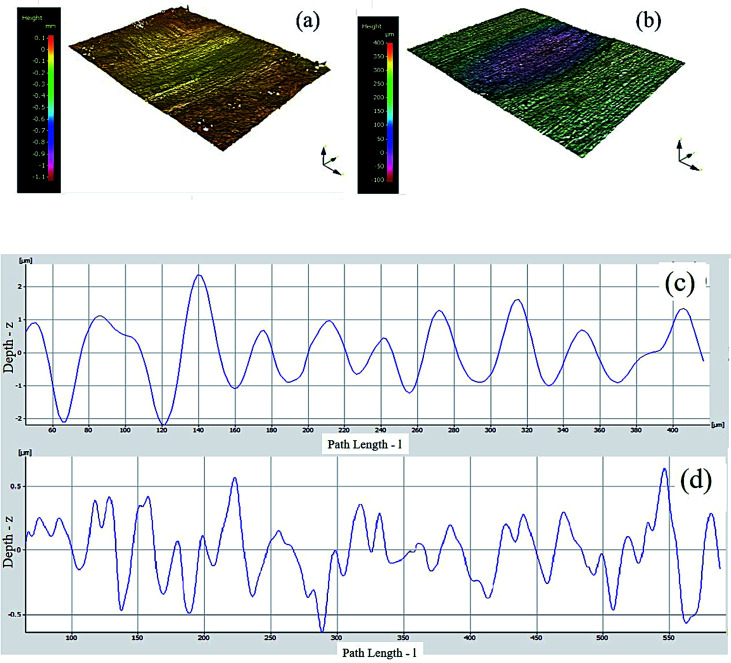
3D surface scans for (a) Al–17Si (b) Ni–WC-15 wt% graphite and surface profilometry scans for (c) Al–17Si (d) Ni–WC-15 wt% graphite.

Fig. 10 identifies the 3D optical surface scans for Al–17Si and Ni–WC-15 wt% graphite worn scars. Visible, in terms of wear depth, the substrate has exhibited comparatively severe wear scar profiles than that of the coatings. However, the severity of wear seems to have reduced, when these samples were coated (Fig. 10(b)). The surface profilometry scans of worn scars were carried out by Mitoyo roughness profilometer for and Al–17Si substrate and HMMC Ni–WC-15 wt%–Gr (Fig. 10). The depth of grooves can be observed in the form of peaks and valleys. From the surface profilometry across the scars as shown in Fig. 10(c) and (d), the maximum groove depth inside the wear scar between pile-ups is approximately 2.8 μm for Al–17Si and the least wear depth is observed for Ni–WC-15 wt%–Gr (0.7 μm). Hirschhron & Daver^29^ in their research on powder metallurgy of Ni–WC–Gr coating mentioned that as the amount of graphite in increases, a reduction in surface roughness and width of wear grooves is witnessed.

Table 1 presents a summary of tribo-mechanical properties of the substrate and coatings that were obtained in the current research. A comparative evaluation is necessary for determining the degree of effectiveness of graphite doping on wear resistance of HMMC coatings and the change in mechanisms due to which the tribological properties were enhanced. Under the dry sliding conditions, the majority wear mechanisms that have been identified in the current research, are categorized as oxidative, abrasive, adhesive, delamination, extrusion or melting and fatigue induced wear. In general, it is quite evident that the base alloy has undergone intense abrasion on the substrate along with adhesion on counter-bodies with greater material removal in the form of delaminated debris. Interesting results were comprehended when 5 wt% of graphite was added in Ni–WC coating deposited on Al–Si alloy. A positive effect on the intensity of wear was observed when the concentration of graphite was further raised and oxidative mild wear was identified as the primary mechanism involved. This was due to the fact that the transfer film generated between the counter face and the sliding area was formed more uniformly and the shearable graphite was able to further reduce the friction.

**Table 1:** A summary of tribo-mechanical properties of substrates and coatings

Tribo-mechanical properties	Surface hardness (*H*_v_)	Surface roughness *R*_a_ after wear (nm)	Wear (mg)	Friction coefficient
**Substrate**				
Al–17Si	85	541	0.7	0.47

**HMMC coatings**				
Ni–WC-5 wt% Gr	781	311	0.33	0.29
Ni–WC-10 wt% Gr	749	256	0.24	0.3
Ni–WC-15 wt% Gr	711	194	0.10	0.21

## 4. Conclusion

In this research work, Ni–WC–Gr coatings were fabricated and deposited on Al–17Si hypereutectic piston alloy by varying concentrations of graphite in the Ni–WC coating. The following conclusions can be drawn herein:

(1) The HMMC coatings of graphite were composed of the WC phase embedded in the Al–Ni intermetallic phase. The intermetallic phase was formed based on the concentration of the nickel in the coating comprising of AlNIi, Al_3_Ni, Al_3_Ni_2_, and Ni_3_Al compounds.

(2) The hardness of graphite based coatings lessened as the concentration increases with a maximum hardness of 781*H*_v_ for Ni–WC-5 wt% graphite.

(3) The optimum concentration of graphite in Ni–WC coating was found to be 15 wt% for acquiring lowest friction coefficient that corresponded to lower wear rates.

(4) The friction coefficient of Al–Si under dry sliding conditions was reduced to from 0.47 to 0.21 with the addition of graphite. The higher reduction in the friction coefficient was attributed to the formation of a shearable transfer layer that prevented delamination and thus, reduced adhesion, abrasion and fatigue cracking. The wear resistance of Al–Si hypereutectic piston alloy was improved 7 times after coatings.

## Conflicts of interest

There are no conflicts to declare.

## Supplementary Material
